# The relationship between trait emotional intelligence and problematic alcohol use among college students

**DOI:** 10.34172/hpp.2022.13

**Published:** 2022-05-29

**Authors:** Robert E. Davis, Nicole A. Doyle, Krishen D. Samuel, Amanda H. Wilkerson, Vinayak K. Nahar

**Affiliations:** ^1^Substance Use and Mental Health Laboratory, Department of Health, Human Performance and Recreation, University of Arkansas, 155 N. Stadium Dr. HPER 308, Fayetteville, AR 72701, USA; ^2^Department of Health Science, 113 Russell Hall, 504 University Boulevard, Box 870313, University of Alabama, Tuscaloosa, AL 35487, USA; ^3^Department of Dermatology, School of Medicine, University of Mississippi Medical Center, Jackson, MS, USA; ^4^Department of Preventive Medicine, School of Medicine, University of Mississippi Medical Center, Jackson, MS, USA

**Keywords:** Alcohol use, Drug, Emotional intelligence, Harmful alcohol consumption, Students

## Abstract

**Background:** Problematic alcohol use among college students is a significant public health concern. Emotional intelligence (EI), or the ability to detect one’s own and others’ emotions and to use this information to direct behavior, is suggested to mitigate problematic alcohol use. The purpose of this study was to examine the relationship between EI and problematic alcohol use among college students while controlling for drug use covariates.

**Methods:** This cross-sectional study utilized an online survey comprised of previously validated measures to determine EI, problematic alcohol use, and drug use among college students from a large, public university in the south-central United States. Regression modeling and independent samples t-test were used to determine the relationship between EI and problematic alcohol use.

**Results:** Problematic alcohol consumption was reported among 27.3% of participants (n=587). In regression modeling, EI demonstrated a significant, protective effect on problematic alcohol use (*b* =-0.050, *P* <0.001, 95% CI: -0.076 – -0.023), when adjusting for important covariates. Independent samples t-test showed that students who screened positive for harmful alcohol use reported significantly lower EI values than those who did not (Mean difference=4.53, *t* =2.98, *P* =0.003, 95% CI: 1.54–7.51).

**Conclusion:** The findings from this study show that problematic alcohol use is prevalent among college students and EI may provide a protective effect against this deleterious behavior. Given the findings observed in this study, university officials should incorporate EI training into the university curriculum, such as in onboarding courses for freshman and transfer students, to target prevention of potentially harmful alcohol consumption and associated negative health impacts.

## Introduction

Alcohol use is common in American society and efforts to moderate use remain a public health priority. National data suggests that nearly 140 million Americans 12 years of age and older report past month alcohol use. Further, approximately half of these consumers are estimated to be binge drinkers (i.e. consumption of large amounts of alcohol in a short time).^1^ Alcohol consumption is associated with deleterious outcomes such as violence, suicide, sexual assault, accidents, reproductive complications, poisonings, cancers, heart disease, mental disorders, and addictive characteristics.^2-7^ Estimates indicate that young adults between the ages of 18-25 exhibit a higher prevalence of current alcohol use (56.3%) than those among younger or older groups, and college students are more likely to display binge drinking behaviors (44.4%) than their young adult counterparts (34.9%).^1,8^

College students are at high-risk for problematic alcohol-related behaviors and associated consequences. Problematic alcohol use herein refers to excessive alcohol consumption and/or consumption that causes physical, psychological, or social harm.^9^ Though general mitigation of alcohol consumption is a concern, efforts to curb problematic alcohol use have been prioritized.^4^ Binge drinking refers to excessive consumption of alcohol in a short time period, which is hazardous and typically results in intoxication. Binge drinking is often quantified as having 4 or more standard drinks (i.e. 12 fl oz of regular beer, 8-9 fl oz of malt liquor, 5 fl oz of table wine, or 1.5 fl oz of distilled spirits)^10^ in a two-hour period for females and having 5 or more drinks during the same time period for males.^11^ Binge drinking is frequent among college students,^8^ and this type of alcohol use is associated with other risk behaviors and problematic outcomes such as sexual assault, intoxicated driving, academic problems, assault, and long-term alcohol dependency issues.^12^

The college environment presents unique factors that may increase college students’ risk for problematic alcohol-related behaviors. The transition period into emerging adulthood presents additional stressors for college students, including learning to live away from established support systems, managing personal finances, maintaining existing relationships, and developing new social relationships.^13,14^ Further, alcohol-related behaviors are engrained into campus cultures, which facilitates alcohol-related problems within these environments.^15^

Individual characteristics including sensation seeking, impulsivity, and extraverted or neurotic personality associate with problematic alcohol use among college students.^16-18^ Further, students often do not possess the necessary coping skills that prevent maladaptive coping behaviors like substance use.^19^ Alcohol use, as means of affective stabilization, increases the risk of alcohol-related problems.^20^ Certain perceptual factors such as outcome-related behavioral and normative beliefs have also been associated with increased alcohol and other substance use among students.^21-23^ For example, alcohol is believed to mitigate social anxiety, provide social stimulus, and lead to satisfying emotional experiences among young people.^24,25^ Additionally, problematic alcohol use is associated with normative beliefs and pressure to conform to perceived norms.^22^ College students often assume that alcohol use is an integral and synchronous part of the college experience,^12^ while often maintaining the inaccurate perception of campus consumption to be much higher than reality, thus leading to heavier drinking.^26^ Such substance-related miss-estimations promote and exacerbate problem drinking.^27^

Emotional intelligence (EI) refers to a set of abilities allowing individuals to accurately detect and interpret their own emotions, as well as the emotions of others as they occur, and use this information to regulate behavior and navigate social settings.^28,29^ EI is positively associated with higher levels of adaptive coping and lower levels of maladaptive coping after controlling for the effects of both general cognitive ability and personality type.^30^ Lower EI has been associated with engagement in risk behaviors among students.^31-33^ More specifically, research suggests that EI associates with substance use^34-36^ inclusive of alcohol.^34,37,38^ It is suggested that greater levels of EI among young people may protect an individual against maladaptive substance-related coping behaviors^30,35,37^and also relate to one’s ability to resist social pressure to engage in alcohol and drug use.^36^ EI has been shown to impact alcohol consumption indirectly by moderating the relationship between social pressure and alcohol consumption,^39^ and to moderate the relationship between perceived alcohol peer norms and alcohol use.^40^ Researchers have also documented that gender moderates the relationship between EI and substance use.^41^ It has also been suggested that EI impacts impulsivity, and may therefore, mediate the relationship between impulsivity and problematic drinking.^18^

Meta-analytic findings show a relationship between alcohol use and EI with a stronger relationship observed between EI and problem alcohol use than between EI and alcohol use frequency.^37^ However, there is a limited body of evidence examining relationships between EI and validated measures of problematic alcohol use and fewer assessing problematic use among students.^30,42^ To our knowledge, no studies among this population have adjusted for the possible confounding effects of drug use when assessing relationships between EI and alcohol use or problematic alcohol use. Dated evidence suggests moderate relationships between EI and alcohol use exist, with stronger relationships existing between EI and problematic alcohol use.^37^ Limited studies have examined EI in relationship to drug use,^34,35^ with conclusions indicating significant relationships.^36,43^ Therefore, the purpose of this study was to examine the relationship between EI and harmful alcohol use in college students utilizing validated measures of both EI and problematic alcohol consumption while controlling for other important covariates, including other drug use.

## Material and Methods

### Participants and procedures

This study utilized a cross-sectional anonymous electronic survey design. Participants in the current study were students at a large, public university located in the south-central United States. These individuals were recruited using non-probability sampling methodology. Participants were recruited, from general education courses as well as through an advertisement in the University’s daily news email. The e-news bulletin is sent daily to every student possessing a valid university email address (approximate University enrollment = 27 000). Further, recruiting from general education courses was undertaken as these classes are not discipline specific and include students from various academic concentration areas (i.e. majors). Inclusion criteria required the participant be at least 18 years of age and currently enrolled in the university. To incentivize participation, students were offered the option of entering a drawing to win one of several gift cards.

### Measures

*Participant characteristics*

Individual characteristics typical of literature focusing on student populations were assessed to describe our sample. Items were included to assess age, gender identity, race/ethnicity, sexual identity, Greek affiliation, academic classification, grade point average (e.g. GPA), employment, and living arrangement (i.e. on campus or off).

*Alcohol use*

To assess alcohol behaviors, students identified if they had consumed alcohol within the last six months (i.e. yes/no) and at what age they first consumed alcohol. Students reported age at onset in years on a 7-point scale; 1 = less than 10, 2 = 10-11, 3 = 12-13, 4 = 14-15, 5 = 16-17, 6 = 18-20, 7 = 21+. Because of data distribution, the age at first consumption was dichotomized to indicate onset prior to age 18. This delineation also indicates, as a pseudo measure, alcohol onset prior to college. Next, students completed the alcohol use disorder identification test (AUDIT). The AUDIT is a 10-item instrument designed to identify problematic (i.e. hazardous or harmful) alcohol use patterns and is indicative of a possible alcohol use disorder.^9^ The AUDIT is not intended to substitute physician intervention or diagnosis; however, it is utilized as a screening tool for problematic alcohol use behaviors in a variety of settings including primary care clinics, emergency rooms, judicial programming, and research settings. This instrument specifically assesses alcohol related domains such as hazardous use, dependence symptoms, psychological harm, and social harm. Sample items include “How often do you have six or more drinks on one occasion?”; “How often during the last year have you felt guilt or remorse after drinking?”; “How often during the last year have you found that you were unable to stop drinking once you started?”; “How often during the last year have you failed to do what was normally expected from you because of drinking?”; “Have you or someone else been injured because of your drinking?”. Items assessing frequency of these alcohol-related behaviors are scored from 0 (“Never or No”) to 4 (“4 times a week or more or Yes, during the last year”). A score of 8 or higher on this instrument is considered a threshold indicative of problematic (i.e. hazardous or harmful) alcohol use. This instrument has demonstrated validity across various age, gender, and cultural settings.^44-46^ In the current study, the AUDIT scale was treated as a continuous measure of alcohol related problems, as well as dichotomized to differentiate between hazardous (score ≥ 8) and non-hazardous (score < 8) drinkers. In the current study, internal consistency for the AUDIT was acceptable (Cronbach’s alpha = 0.83).

*Drug use*

We assessed drug use among participants using a series of items assessing frequency of drug use over a recall period of the previous 6 months. Specifically, marijuana, cocaine, ecstasy, heroin, and methamphetamine use, as well as, prescription drug misuse were assessed. Preceding the prescription drug misuse items, participants were provided a clarifying statement: “The following questions refer to use of prescription medication in a way not specifically directed by a doctor. By this, we mean any use without your own prescription, for recreational purposes, taking higher dose than prescribed, using more frequently than directed, or continued use despite no longer experiencing the problem for which it was prescribed.”^47^ Further, prescription drug items provided examples of specific medications that existed within specific class of drugs, for example, opioids. A sample drug item read “How frequently over the past 6-months have you used prescription opioid medications in a way not specifically directed by a doctor?” and “How frequently have you used marijuana (e.g. cannabis, hashish, pot, grass, THC, etc.) over the past six months?”. Response options for drug items were 1 = “never,” 2 = “1-2 occasions,” 3 = “3-5 occasions,” 4 = “6-9 occasions,” 5 = “10-19 occasions,” 6 = “20-39 occasions,” and 7 = “40 or more occasions.” From responses, dichotomous (yes/no) variables were constructed representing any use/misuse over the past 6-months.

*Emotional intelligence*

A 33-item valid and reliable scale was used to measure trait EI.^48^ The Schutte Emotional Intelligence Scale assesses the individual’s ability to interpret and regulate their own emotions as well as the emotions of others using a uni-factorial structure. Sample items from this scale read “I am aware of my emotions as I experience them”, “I find it hard to understand the non-verbal messages of other people”, “I have control over my emotions”, “I recognize my emotions as I experience them”, and “It is difficult for me to understand why people feel the way they do”. Response options for each item exist on a 5-point agreement scale 1 = “strongly disagree,” 2 = “somewhat disagree,” 3 = “neither disagree nor agree,” 4 = “somewhat agree,” 5 = “strongly agree,” with several items reverse coded. In the current study, internal consistency of the EI scale was strong (Cronbach’s alpha = 0.88).

### Statistical analysis

Descriptive statistics in the form of means, standard deviations (SD), frequencies and percentages were calculated to define the characteristics of the study sample. In order to examine the relationship between EI and problematic alcohol behavior, multiple analyses were conducted. First, regression modeling was utilized followed by group testing in the form of independent samples *t*test. In addition to EI, the regression model was constructed to include covariates that exhibited a significant univariate relationship with AUDIT scores. For the regression procedures, EI and GPA were continuous predictors with the substance use variables treated dichotomously. To further understand the relationship between EI and harmful alcohol use, a *t*test was employed to examine differences in mean EI scores between those engaging in harmful alcohol behavior and those who did not. Group delineation was based on the established AUDIT cut point of 8, with those equaling or exceeding the cut point classed as hazardous alcohol use. An a priori alpha level of 0.05 was established to indicate significant differences in testing. Missing data was handled using listwise deletion. All analyses were conducted in IBM SPSS Statistics version 24.0 Armonk, NY: IBM Corp. G*Power version 3.1, Aichach, Germany was used to estimate sample size requirements for regression modelling. Alpha was set to 0.05, power at 0.80, number of predictors at 10, with effect size set to 0.10 (small-medium) dictated a minimum sample of 172 participants. Because model building would be based on inclusion of only those covariates exhibiting significant bivariate relationships to AUDIT scores (the dependent variable), predictors for the power analysis were set at 10 to be conservative.

## Results

As seen in Table 1, the mean age of participants (*N* = 587) was 22 years of age. The majority of individuals identified as female (70.9%), White/Caucasian (74.6%), heterosexual (85.2%), and not affiliated with a Greek society (73.3%). Barring freshman (8.9%), representation in the study was equitable across academic classification. The majority of students lived off campus, and 59.3% reported some kind of employment in addition to being a student.

**Table 1:** Descriptive Statistics (n = 587)

**Variables**	
Age, Mean (SD)	22.63 (6.10)
Grade point average, Mean (SD)	3.57 (0.40)
Emotional Intelligence, Mean (SD)	129.61 (14.37)
AUDIT^a^ score, Mean (SD)	5.99 (5.39)
Gender identity, No. (%)	
Female	416 (70.9)
Male	157 (26.7)
Sexual identity, No. (%)	
Heterosexual	500 (85.2)
Non-heterosexual	77 (13.1)
Race/Ethnicity, No. (%)	
White/Caucasian	438 (74.6)
Other	140 (23.9)
Greek affiliation, No. (%)	
Non-Greek	430 (73.3)
Fraternity/Sorority	147 (25.0)
University status, No. (%)	
Freshman	52 (8.9)
Sophomore	120 (20.4)
Junior	141 (24.0)
Senior	135 (22.8)
Graduate student	106 (18.1)
Living status, No. (%)	
On campus	132 (22.5)
Off campus	440 (75.0)
Employment status, No. (%)	
Employed	348 (59.3)
Unemployed	223 (38.0)
Problematic alcohol use, No. (%)	
Non-problematic	338 (57.6)
Problematic	160 (27.3)
Alcohol onset, No. (%)	
< 18 years	302 (51.4)
≥ 18 years	259 (44.1)
Marijuana, No. (%)	
No use	387 (65.9)
Marijuana use	194 (33.0)
Illicit Drug use^b^	
No use	539 (91.8)
Drug use	37 (6.3)
Prescription drug misuse^c^	
No Misuse	491 (83.6)
Misuse	89 (15.2)

Percentages reported may not equal 100% due to participant omission of data. ^a^Alcohol use disorder identification test. ^b^Illicit drug use (other than marijuana & prescription drug misuse). ^c^Indicates misuse of prescription drugs (i.e. opioids, sedatives, tranquilizers, or stimulants).

Among this sample, alcohol use was prevalent, as only 15.5% (0.155, 95% CI: 0.125 – 0.185) of participants reported no alcohol consumption over the past 6 months. The mean AUDIT score was 5.99 (SD = 5.39) (Table 1). Further, 27.3% of the sample screened positive for problematic alcohol consumption (score ≥ 8) based on the validated AUDIT instrument. Marijuana use, prescription drug misuse, and other illicit drug use was reported by 33.0%, 15.2%, and 6.3% of participants, respectively.

To investigate the relationship between EI and problematic alcohol use we regressed AUDIT scores onto EI in the presence of important covariates (Table 2). Included covariates were chosen due to their significant univariate relationships to AUDIT scores. The model collectively explained roughly 40.0% of the variance in AUDIT scores (*R*^2^ = 0.395, *P* < 0.001). Importantly, EI exhibited a significant protective effect on AUDIT score (*b*= -0.050, *P* < 0.001), whereas higher EI was associated to lower AUDIT scores. In this modeling, other illicit drug use (*b* = 3.722, *P* < 0.001), marijuana use (*b* = 2.273, *P* < 0.001), prescription drug misuse (*b* = 4.188, *P* < 0.001), alcohol use prior to age 18 (*b*= 1.243, *P* = 0.002), identifying as male (*b* = 1.357, *P* < 0.001) and being affiliated with a fraternity or sorority (*b* = 1.535, *P* < 0.001) were associated with increased AUDIT scores, representing more problematic alcohol use. When examining the standardized regression coefficient of EI in relation to those of other variables, it becomes apparent that the protective effect of EI may be considerably impactful.

**Table 2:** Multiple regression of alcohol use disorder identification test (AUDIT) score onto predictor variables

	**b**	**SE**	**B**	**LBCI**	**UBCI**	**P value**
Emotional intelligence^a^	-0.050	0.013	-0.130	-0.076	-0.023	< 0.001
Illicit drug use	3.722	0.802	0.177	2.146	5.298	< 0.001
Marijuana use	2.273	0.444	0.201	1.402	3.144	< 0.001
Prescription drug misuse	4.188	0.567	0.287	3.074	5.303	< 0.001
Alcohol use prior to age 18	1.243	0.404	0.114	0.450	2.037	0.002
Gender	1.357	0.376	0.128	0.619	2.095	< 0.000
Greek affiliation	1.535	0.428	0.126	0.695	2.376	< 0.001
Grade point average^a^	-0.874	0.483	-0.064	-1.822	0.075	0.071
Model:* R*^2^ = 0.395, *F* = 41.529, *P*< 0.001						

^a^Continuous variable. Reference categories for dichotomous predictors: substance variables = no use; gender = female; Greek = non-Greek; Alcohol use prior to age 18 = 18+ years. SE = standard error of the estimate; LBCI= lower bound of the 95% confidence interval for b; UBCI = upper bound of the 95% confidence interval for b.

Further, we examined statistically significant differences in EI between those exhibiting problematic alcohol use and those who did not. As expected, those who screened positive for problematic alcohol use had significantly lower EI scores (M = 126.85) compared to those who did not exhibit such alcohol-related characteristics (M = 131.38, *t* = 2.98, *P* = 0.003) (Table 3).

**Table 3:** Mean differences in emotional intelligence between problematic and non-problematic drinkers

	**n**	**Mean**	**SD**	**Mean difference**	**t**	***P*** ** value**	**Effect size**
Problematic drinking	160	126.85	17.02	4.53	2.98	0.003	0.300
Non-problematic drinking	338	131.38	12.87				

Problematic or harmful drinking identified by the alcohol use disorder identification test (AUDIT) using an established cut point (≥8). Effect size is measured as Cohen’s *d*.

## Discussion

The purpose of the current study was to examine the relationship between EI and problematic alcohol use among college students, while adjusting for important demographic and substance use covariates. Alcohol use is prevalent in society and on college campuses^1,15,49^ with students reporting higher levels of problematic drinking^8^ than their age-matched, non-student counterparts.^1^ Students are in a unique environment that forces them to manage relationships, academics, and finances, among a multitude of other stressors.^13,14^ This can be particularly challenging given the potential lack of coping skills.^19^ Therefore, the importance of assessing EI and problematic drinking behaviors while accounting for other important covariates is a key step in better understanding these relationships in college students. Herein, we found illicit drug use, marijuana use, prescription drug misuse, alcohol consumption before age 18, identifying as male, and being a member of Greek organization to significantly associate with increased problematic alcohol use, as identified by the AUDIT. Importantly, higher EI scores were significantly associated with lower AUDIT values. Further, those who exceeded the established cut point indicative of problematic drinking using the AUDIT exhibited significantly lower EI values than those beneath this threshold. Thus, our findings suggest EI as a protective factor related to problematic alcohol use.

In the current study, 85% of students reported drinking in the past 6 months, with 27% exhibiting problematic and potentially harmful patterns of drinking behavior, as defined by the AUDIT screening tool. The AUDIT is a validated instrument that assesses patterns and behaviors associated with problematic alcohol use. The cut-point indicative of problematic alcohol use is a score of 8 or higher. The current sample reported a mean AUDIT score of 5.99 (SD = 5.39). Kokotailo and colleagues reported a mean AUDIT score of 7.00 for their college student sample (n= 302). Their sample consisted of students who were accessing services at a student health clinic, and their mean AUDIT scores may be higher due to this reason.^50^ In a 2012 study conducted by DeMartini and Carey, the authors used the AUDIT to screen for potential problematic drinking and found that 207 out of 401 (51.6%) of their college student sample screened positively for this behavior; the current study found 158 out of a sample of 587 (27%) screened positively for problematic drinking.^51^ DeMartini and Carey’s sample consisted of 69% freshman, whereas the current study consisted of approximately 9% freshman. Using the AUDIT, Claros and Sharma reported just 11% of their sample of 199 community college students screened positive for problematic alcohol use.^34^ This finding in comparison to those of the current study as well as findings of Kokotailo et al^50^ and DeMartini and Cary^51^ may indicate socio-structural differences between community college and larger, four-year university environments, as it pertains to alcohol behavior. These discrepant participant characteristics likely relate to proportional differences in positive screens for problematic drinking.

It has been suggested that lower cut-points for college student populations should be applied to the AUDIT to better assess problematic behavior. In theory, lower cut-points could better inform targeted intervention strategies, as these lower cut-points could potentially aid in identification of problematic drinking patterns before they reach higher, more deleterious levels.^52^ Given the importance of validated instruments such as the AUDIT, use of this screening tool for student alcohol use behavior has been endorsed by many studies. Continued use, and refinement of, this tool among collegiate populations is encouraged for consistency in this body of literature.

EI has been shown to exhibit protective effects against negative life events,^53^ be associated with higher levels of adaptive coping, and moderate the effects of psychological concerns such as stress, anxiety, and depression.^30^ Additionally, greater levels of EI may protect against maladaptive substance-related coping behaviors and assist with resisting peer pressure regarding alcohol and substance use.^34,37^ Specifically, higher levels of EI allow for greater self-control and emotional regulation, leading to improved decision-making and a higher likelihood of utilizing adaptive coping mechanisms.^18^ It is important to note that alcohol use is often used as a means of affective stabilization (i.e. coping with negative affects and/or situations) which then can manifest into alcohol-related problems.^20^ Therefore, it is posited that higher levels of EI mitigate these negative effects and lead to the use of adaptive coping strategies, which our findings support.

The present study found that students who screened positive for problematic alcohol use exhibited lower levels of EI than those who did not screen positive; mean difference of 4.53. Moreover, this mean difference was significant (*t* = 2.98, *P* = 0.003). Thus, those with higher EI scores were less likely to exhibit problematic alcohol use than those with lower EI scores. Therefore, our study provides support for the hypothesis that EI associates with alcohol use and should be considered a protective factor against problematic alcohol use. Further, using regression modeling we found EI to be a significant predictor of AUDIT scores. Notably, this relationship remained significant (*b* = -0.050, *P* < 0.001) even in the presence of several known correlates of alcohol use. In total, our model explained approximately 40% of the variation in AUDIT scores. Examination of the standardized regression coefficient of EI (-0.130), indicates that EI contributes valuably to the understanding of problematic alcohol use among this sample of students. A strength of the current study was that we adjusted the relationship between EI and problematic alcohol use for substance use covariates. Authors of a meta-analytic investigation suggest a lack of existing literature accounting for other substances in their investigations of relationships between EI and alcohol use and advocate for future research to address this limitation.^37^

There is ongoing discussion in the literature^54^ regarding whether EI should be measured as an ability (i.e. with performance-like tests) or as a more stable trait, which refers to the propensity to act similarly across situations (i.e. measured with questionnaires as we have done). In most instances, EI researchers opt for the latter, viewing EI as a consistent set of emotional responses across different contexts. Specific to alcohol use, meta-analytic findings suggest that the relationship between EI and alcohol involvement does not differ significantly based on trait or ability measures of EI.^37^ Potentially, a more important question is whether EI can be increased through training, and if so, what are important consequences of improved EI? A meta-analysis by Kotsou et al examined studies that looked at both measurements of EI as an ability and as a trait, outcomes of interventions to increase EI, and the subsequent effect an increase in EI had on various domains. They found mixed results for studies that measured EI as an ability. Of 37 studies using trait EI measures, however, 32 found a significant increase in EI after EI training.^54^ Importantly, of 18 studies that specifically looked at the effects of increased EI on psychological and physical health, 16 reported significant positive results, indicating the protective effects of increased EI on both psychological and physical health outcomes.

Research has shown a consistent relationship with alcohol consumption and factors such as impulsivity, with greater impulsivity and disinhibition associated with increased alcohol consumption.^55,56^ Specifically, those with high urgency and sensation-seeking tendencies display higher levels of alcohol consumption and its related problems.^16^ It has also been suggested that higher levels of EI may reduce impulsivity and therefore mediate the relationship between impulsivity and problematic alcohol use.^18^ This warrants further investigation of impulsivity and its relationship with problematic alcohol use, as well as the potentially protective effects higher EI could have in mediating this relationship.

While our study utilized a uni-factorial EI scale, different underlying aspects of EI may require attention when considering interventions, such as the underlying ability for emotional competence, typical performance of EI, and emotional self-efficacy.^57^ It may be most beneficial to use a multi-factorial scale for measurement and to inform future interventions, such as the Trait Meta-Mood Scale (TMMS-24)^58^ which considers three dimensions of EI: emotional attention (i.e. ability to identify one’s emotions and those of others and know how to express them), emotional clarity (i.e. understanding of emotions), and emotional repair or regulation (i.e. ability to handle emotions). Recent research conducted with college students in Spain by Merchán-Clavellino et al highlights the role that emotional clarity plays in mediating alcohol consumption.^18^ Thus, future university-based interventions may need to focus on improving specific underlying aspects of EI and their specific impacts upon problematic alcohol consumption, in order to be most effective.

### Implications for Practice

Findings presented in this study provide support for the relationship between EI and problematic alcohol use among college students. As the body of literature continues to strengthen support for this relationship, including relationships between EI and psychological dysregulation (another common correlate of problematic alcohol consumption)^59,60^ and the ability to increase EI through intervention,^57,61^ we advocate college leadership to consider incorporation of EI focused intervention into programmatic curriculum. Many colleges have required onboarding courses (e.g. University perspectives, Freshman experience) designed to increase the potential for the adaptive transition of incoming freshman and transfer students into their new campus environments. These may be optimal settings to offer brief or longitudinal education or skills-based interventions among students. Evidence based strategies are vast and primarily focus on knowledge and behavioral skills. For instance, strategies serving to increase knowledge regarding emotions, emotion identification skills, emotional facilitation of thought, reflection sessions linking recent desirable and undesirable behaviors to concurrent emotional states, mindfulness-based self-monitoring of and reflection on emotions, self-awareness and detachment, empathy training, among other interventions may increase EI.^57,61^ Universities may also consider EI training among upper administration, faculty, and staff members who directly interact with students. For example, Schutte and colleagues propose that EI development among leadership within social systems enhances functioning of and within the social system.^57^

This study possesses several limitations that the authors would like to acknowledge. First, the cross-sectional research design allows only for speculation of causal linkage between variables. Second, this study used an electronic and anonymous means of data collection which potentiates various biases such as, social facilitation, acquiescence, and recall bias. However, because of the sensitive nature of this study’s topic, our methodology has potential benefits. For example, it is suggested that the AUDIT being administered as a questionnaire rather than an oral interview can save time, costs, and potentially produce more accurate responses from the participant.^62^ Next, the crude nature of our ‘other drugs’ variable could be perceived as a limitation of its utility. However, this style of categorization is not uncommon in the literature when representation of certain illicit drug use (e.g. heroin, methamphetamine, MDMA) is minimal among the study sample.^63^ Moreover, our sample was incentivized to participate with the chance of winning one of several gift cards which may lead to selection bias. Finally, the sample was recruited from a single large US university which my impact generalization of findings.

## Conclusion

The purpose of the current study was to examine a potential relationship between EI and problematic alcohol use among college students. Importantly, EI was significantly and negatively associated with problematic alcohol use after adjustment for important sociodemographic and substance use covariates. Moreover, those who screened positive for problematic alcohol use exhibited significantly lower EI scores using validated instrumentation. With 27% of the sample screening positive for problematic alcohol use, there is a clear need for informed resources and targeted intervention strategies. Given the protective nature of EI observed within our study findings, the authors recommend EI training be incorporated into university curricula.

## Acknowledgements

The authors would like to thank those individuals who participated in this research for their contribution.

## Authors’ contributions

RED and VKN conceptualized the current research and methodologic design. RED, VKN and NAD developed the instrumentation. RED and NAD collected all data. RED, VKN, AHW and NAD are responsible for interpretation of data. All named authors contributed to the drafting of this manuscript and are responsible for intellectual content. All named authors have approved of this manuscript in its current form.

## Funding

No external funding sources were used in conducting the current research.

## Ethical approval

All procedures were approved by the University of Arkansas’ ethics committee (protocol #: 1901169203).

## Competing interests

The authors declare no conflicts of interest.
